# Mutual influence between current-induced giant magnetoresistance and radiation-induced magnetoresistance oscillations in the GaAs/AlGaAs 2DES

**DOI:** 10.1038/s41598-017-05351-8

**Published:** 2017-07-11

**Authors:** R. L. Samaraweera, H.-C. Liu, Z. Wang, C. Reichl, W. Wegscheider, R. G. Mani

**Affiliations:** 1 Department of Physics and Astronomy, Georgia State University, Atlanta, Georgia 30303 USA; 2 Laboratorium für Festkörperphysik, ETH Zürich, CH-8093 Zürich, Switzerland

## Abstract

Radiation-induced magnetoresistance oscillations are examined in the GaAs/AlGaAs 2D system in the regime where an observed concurrent giant magnetoresistance is systematically varied with a supplementary dc-current, *I*_*dc*_. The *I*_*dc*_ tuned giant magnetoresistance is subsequently separated from the photo-excited oscillatory resistance using a multi-conduction model in order to examine the interplay between the two effects. The results show that the invoked multiconduction model describes the observed giant magnetoresistance effect even in the presence of radiation-induced magnetoresistance oscillations, the magnetoresistance oscillations do not modify the giant magnetoresistance, and the magnetoresistance oscillatory extrema, i.e., maxima and minima, disappear rather asymmetrically with increasing *I*_*dc*_. The results suggest the interpretation that the *I*_*dc*_ serves to suppress scattering between states near the Fermi level in a strong magnetic field limit.

## Introduction

The realization of novel photo-excited zero-resistance states and 1/4–cycle shifted magnetoresistance oscillations in the high quality GaAs/AlGaAs two-dimensional electron system by low energy photons in the low magnetic field, high filling factor limit^1–3^ has led to new interest in the experimental^1–39^ and theoretical^40–62^ study of photo-excited transport in low dimensional systems. The same high quality GaAs/AlGaAs two-dimensional electron system (2DES) has also served to provide new insights into an observable giant magnetoresistance in the 2DES^63–81^. Here, for example, recent studies have shown remarkable features such as size dependence and simple tunability of the GMR with a supplemental dc-current (*I*_*dc*_)^77, 80^. The observability of these two-interesting effects over the same range of magnetic fields and temperatures has created experimental interest into the questions related to the mutual influence between the photo-excited oscillatory magnetoresistance and the *I*_*dc*_ induced GMR in the 2DES and whether possible mutual influence can be utilized to obtain new physical insight into the two effects. Thus, we examined the overlap of microwave photo-excited magnetoresistance oscillations^1–14, 16–19, 21, 22, 24, 25, 28–30, 33–38^ with a *I*_*dc*_-induced positive and negative-GMR in the GaAs/AlGaAs 2DES^77, 79–81^. The aims of this study were (a) to separate, if possible, the two effects when they occur together, using an empirically established multi-term Drude conduction model^77, 80^, in order to extract the characteristics of each individual effect, and (b) to then evaluate and determine possible mutual influence between the two effects. The results confirm separability of the two effects and show, in addition, that the GMR inducing *I*_*dc*_ serves to suppress the peaks (maxima) in the photo-excited magnetoresistance oscillations while leaving the valleys (minima) of the microwave induced oscillations relatively unaffected.

## Results

**Figure 1:**
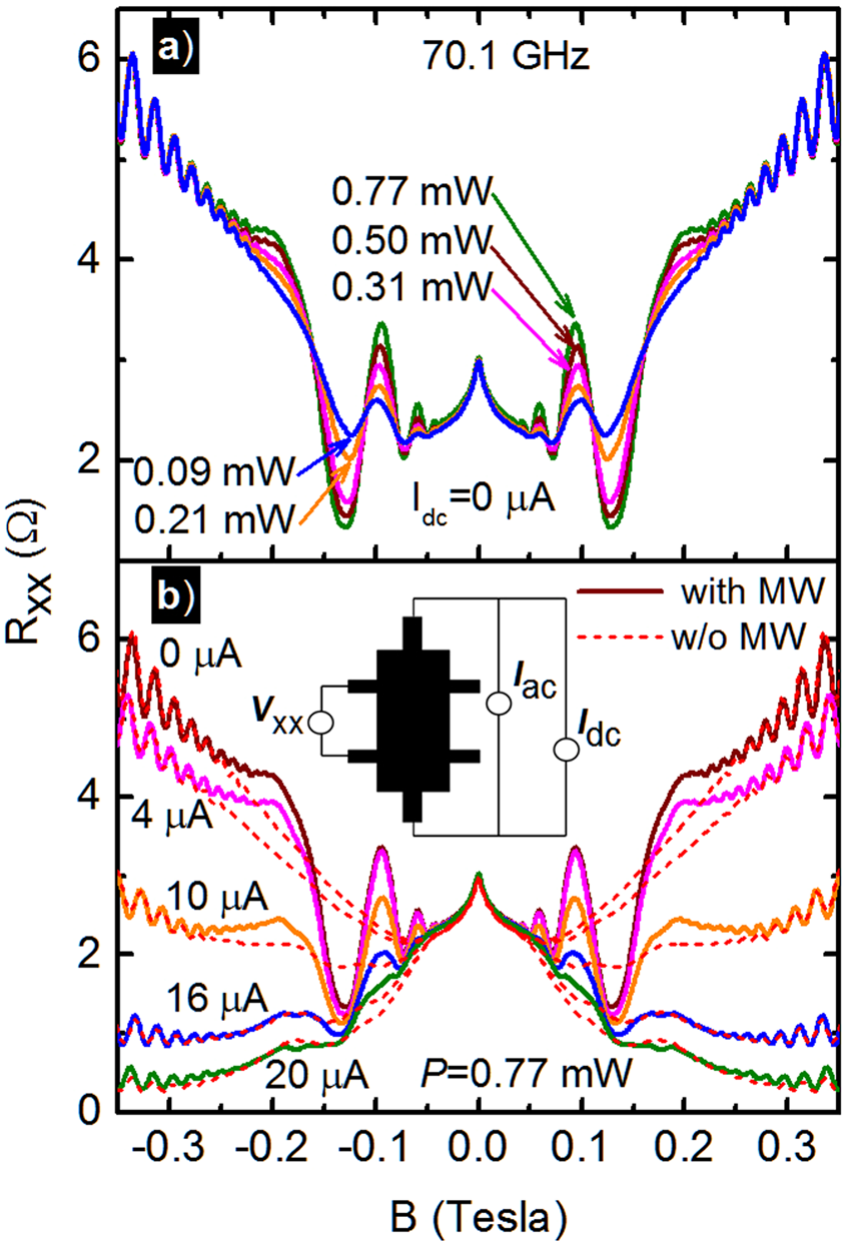
Radiation induced magnetoresistance oscillations in *R*_*xx*_ in a GaAs/AlGaAs hetreostructure 2D electron system. (**a**) *R*_*xx*_ is exhibited vs. the magnetic field, *B*, at different microwave power, *P*, for 0.09 ≤ *P* ≤ 0.77 *mW* and *I*_*dc*_ = 0 *μA*. (**b**) This panel exhibits *R*_*xx*_ vs. *B*, at different *I*_*dc*_ for 0 ≤ *I*_*dc*_ ≤ 20 *μA*, with microwave excitation at *f* = 70.1 GHz and *P* = 0.77 *mW* (solid lines), and under dark conditions (dashed lines).

Figure 1(a,b) exhibit the magnetoresistance of a GaAs/AlGaAs Hall bar specimen under *f* = 70.1 *GHz* photoexcitation at *T* = 1.7 *K*. Figure 1(a) shows the diagonal resistance *R*_*xx*_ vs. the magnetic field, *B*, for various microwave source power levels, *P*. The non-oscillatory portion of the data show initial negative magnetoresistance to *B* = 0.15 *T*, followed by positive magnetoresistance to *B* = 0.35 *T*, with observable Shubnikov-de Haas oscillations for *B* ≥ 0.2 *T* ^25, 28^. The data also confirm, that in the standard photo-excited experiment, the radiation-induced magnetoresistance oscillations, which are observed roughly over the interval −0.2 ≤ *B* ≤ 0.2 *T*, increase in amplitude with increasing *P* upto *P* = 0.77 *mW*. The inset of Fig. 1(b) illustrates the configuration, which is the principal focus of this study, for the transport measurements carried out with a supplementary dc-current, *I*_*dc*_ in order to examine the influence of the *I*_*dc*_^80^ on both the non-oscillatory- and photo-excited oscillatory magnetoresistance in this system. Thus, the main panel of Fig. 1(b) exhibits the *R*_*xx*_ under constant photo-excitation of *f* = 70.1 *GHz* at *P* = 0.77 *mW* (solid lines) and in the dark (red-dashed-lines) for various values of *I*_*dc*_ over the span 0 ≤ *I*_*dc*_ ≤ 20 *μA*. A detailed study of the influence of *I*_*dc*_ on *R*_*xx*_ under dark conditions appears elsewhere^80^. Figure 1(b) shows that the non-oscillatory magnetoresistance below *B* = 0.05 *T* is uninfluenced by the *I*_*dc*_ while the magnetoresistance for 0.05 ≤ *B* ≤ 0.35 *T* changes from an overall positive magnetoresistance to an overall negative giant magnetoresistance with increasing *I*_*dc*_. That is, a negative magnetoresistance effect develops with increasing *I*_*dc*_ at *B* ≥ 0.05 *T*, which reduces the *R*_*xx*_ by nearly 40 percent at 0.10 *T*. Note that the |*B*| ≤ 0.05 *T* magnetoresistance, which is unaffected by both *P* and *I*_*dc*_, appears similar in shape to the weak-localization effect^77, 78^. The figure also shows clearly that the amplitude of the photo-excited magnetoresistance oscillations decreases with increasing *I*_*dc*_.

Since one aim of the study was to characterize the change in the radiation-induced magnetoresistance oscillations produced by the supplementary *I*_*dc*_ in order to gain further understanding of the physical effect of the *I*_*dc*_, we worked to separate out the non-oscillatory and oscillatory terms in the observed *R*_*xx*_. Thus, we introduced a fitting model that addressed both the weak localization like- and the “bell shape” giant magnetoresistance- terms. The weak localization like magnetoresistance was accounted for by including a term ${R}_{xx}=A\,{ln}({B}_{0}/B)$, which is what would be expected from 2D WL theory upon neglecting spin-orbit and spin scattering^82, 83^. The GMR effect was addressed with the multi-conduction Drude model^77^. Thus, we set ${R}_{xx}={\rho }_{xx}(L/W)$, where, *ρ*_*xx*_ is the diagonal resistivity, and *L*/*W* is device length-to-width ratio. For the sample studied, *L*/*W* = 1, which sets *R*_*xx*_ = *ρ*_*xx*_. The diagonal resistivity, *ρ*_*xx*_ and off-diagonal resistivity, *ρ*_*xy*_ were expressed in terms of diagonal conductivity, *σ*_*xx*_ and off-diagonal conductivity, *σ*_*xy*_ by ${\rho }_{xx}={\sigma }_{xx}/[{\sigma }_{xx}^{2}+{\sigma }_{xy}^{2}]$ and ${\rho }_{xy}={\sigma }_{xy}/[{\sigma }_{xx}^{2}+{\sigma }_{xy}^{2}]$. Since the experimental results indicate that the GMR changes with *I*_*dc*_, see Fig. 1(b), the conductivities include two terms: ${\sigma }_{xx}={\sigma }_{xx}^{0}+{\sigma }_{xx}^{1}={\sigma }_{0}/\mathrm{[1}+{({\mu }_{0}B)}^{2}]+{\sigma }_{1}/\mathrm{[1}+{({\mu }_{1}B)}^{2}]$. (Similarly, ${\sigma }_{xy}={\sigma }_{xy}^{0}+{\sigma }_{xy}^{1}={\mu }_{0}{\sigma }_{0}B/\mathrm{[1}+{({\mu }_{0}B)}^{2}]+$ ${\mu }_{1}{\sigma }_{1}B/\mathrm{[1}+{({\mu }_{1}B)}^{2}]$). The terms ${\sigma }_{xx}^{1}$ and ${\sigma }_{xy}^{1}$ account for the changes in the conductivity due *I*_*dc*_ and *P*. Note that ${\sigma }_{xy}^{1}={\sigma }_{xx}^{1}\times {\mu }_{1}B$. The zeroth conductivity terms, ${\sigma }_{xx}^{0}$ and ${\sigma }_{xy}^{0}$ represents the high mobility electrons in 2D-electron system. Thus, *σ*_0_ = *n*_0_ *eμ*_0_, where *n*_0_ is the electron density and *μ*_0_ is the electron mobility in the 2D electron system. The multi-conduction model with two conductivity terms includes four parameters *μ*_0_, *σ*_0_, *μ*_1_ and *σ*_1_. However, the number of free parameters has been reduced to two, i.e. *μ*_1_ and *σ*_1_, by holding constant *n*_0_ and *μ*_0_ to the values extracted from the low field dark measurements at *I*_*dc*_ = 0. With such accounting for both the weak localization-like magnetoresistance and the bell-shape giant magnetoresistance, the experimental magnetoresistance data were fit to ${R}_{xx}=A\,ln({B}_{0}/B)+{\sigma }_{xx}/[{\sigma }_{xx}^{2}+{\sigma }_{xy}^{2}]$.

**Figure 2:**
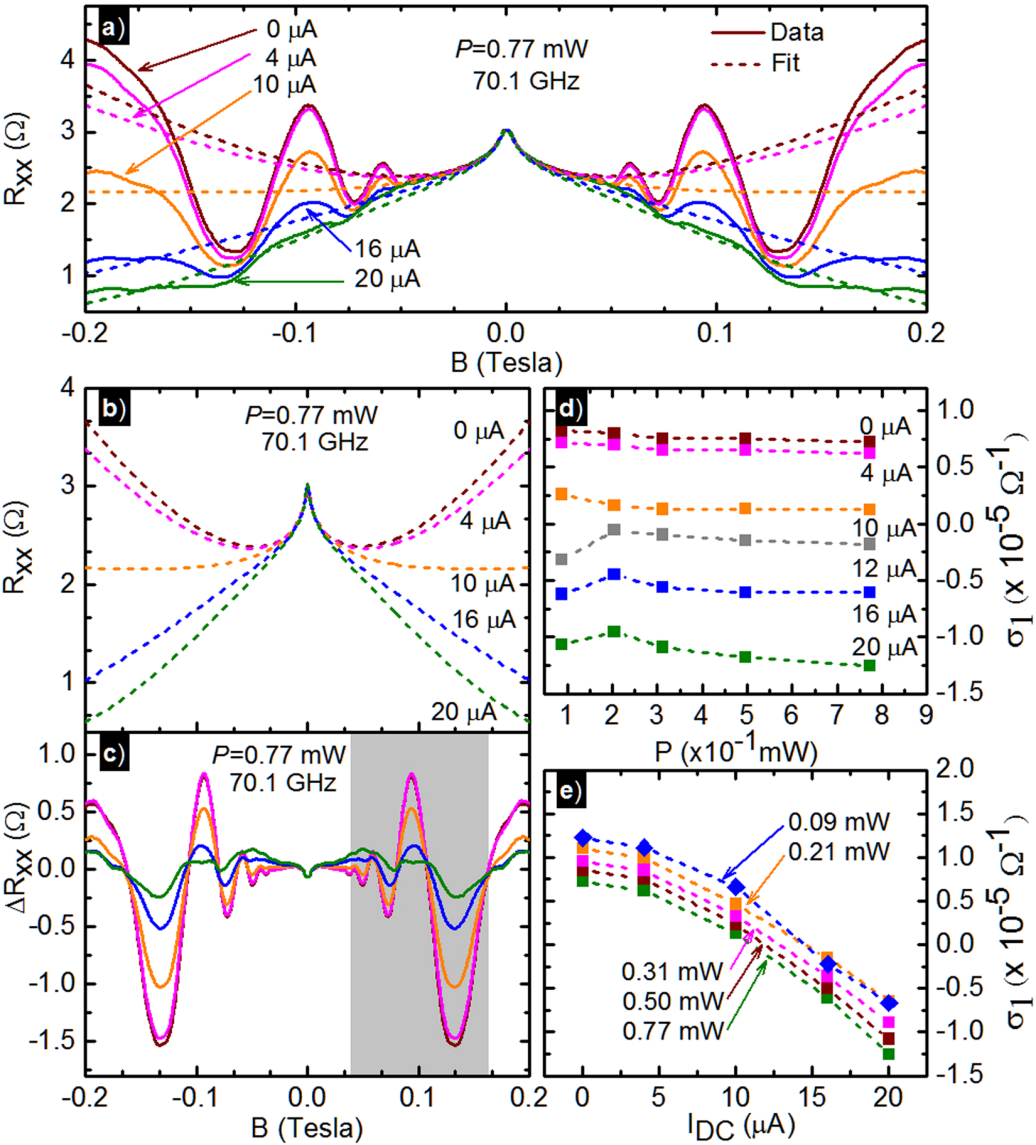
(**a**) Experimental data of Fig. 1(b) with model fits of the non-oscillatory giant magnetoresistance in the GaAs/AlGaAs 2DES using the multi-conduction model described in the text. (**b**) Fit extracted non-oscillatory *R*_*xx*_ of panel (a) at various dc-current, *I*_*dc*_ at *P* = 0.77 *mW*. (**c**) This panel exhibits the extracted radiation-induced magnetoresistance oscillations after subtracting the model fits from the experimental data of panel (a), at various *I*_*dc*_ at *P* = 0.77 *mW*. The highlighted region was used for the fits shown in Figs 3(a) and 4(a). (**d**) Model extracted conductivity *σ*_1_ vs. *P*, at various *I*_*dc*_. (**e**) Shows *σ*_1_ vs. *I*_*dc*_ at different *P*, the traces are offset vertically by $1\times {10}^{-6}\,{{\rm{\Omega }}}^{-1}$ for clarity. Dashed lines in (**d**,**e**) are guides to the eye.

Figure 2(a) exhibits model fits of the non-oscillatory portion of the magnetoresistance data at different *I*_*dc*_, under photoexcitation at *f* = 70.1 *GHz* and *P* = 0.77 *mW*. Figure 2(b) shows the fit extracted non-oscillatory magnetoresistance. Figure 2(c) exhibits the residual resistance Δ*R*_*xx*_ after subtracting the best fit non-oscillatory magnetoresistance from the experimental data, i.e., ${\rm{\Delta }}{R}_{xx}={R}_{xx}-[A\,ln({B}_{0}/B)+{\sigma }_{xx}/[{\sigma }_{xx}^{2}+{\sigma }_{xy}^{2}]]$. Table 1 summarizes fitting parameters for the fits shown in Fig. 2(a,b). For a given *I*_*dc*_, the non-oscillatory magnetoresistance can be fit with a constant *μ*_1_ over the entire *P*-range. Figure 2(d,e) exhibit the fit extracted *σ*_1_, which captures the behavior of the non-oscillatory giant magnetoresistance in the experimental data. As observable in Fig. 2(d), the *σ*_1_ is not significantly affected by the applied *P*. In contrast, in Fig. 2(e), the *σ*_1_ shows a large change as a function of *I*_*dc*_. One can observe a clear transition of *σ*_1_ from positive- to negative- values with increasing dc-bias from 0 *μA* to 20 *μA*. The sign-change in *σ*_1_ occurs in the range of 10 ≤ *I*_*dc*_ ≤ 12 *μA*. Figure 1(b) suggests that the crossover from positive- to negative- magnetoresistance, if one neglects the weak localization like term, occurs over roughly this *I*_*dc*_ interval.

**Table 1:** Parameters extracted from fits of non-oscillatory giant magnetoresistance in Fig. 2(a) at various *I*_*dc*_, see text.

*I*_*dc*_ (*μA*)	*n*_0_ (10^11^ *cm* ^−2^)	*μ*_0_ (10^6^ *cm* ^2^/*Vs*)	*μ*_1_ (10^6^ *cm* ^2^/*Vs*)
0	2.4	11.4	0.019
4	2.4	11.4	0.021
8	2.4	11.4	0.036
12	2.4	11.4	0.041
16	2.4	11.4	0.045
20	2.4	11.4	0.062

Parameters *n*_0_, *μ*_0_ and *μ*_1_ were held constant as a function of *P* at each *I*_*dc*_. The parameter *σ*_1_ has been plotted in Fig. 2(d,e).

**Figure 3:**
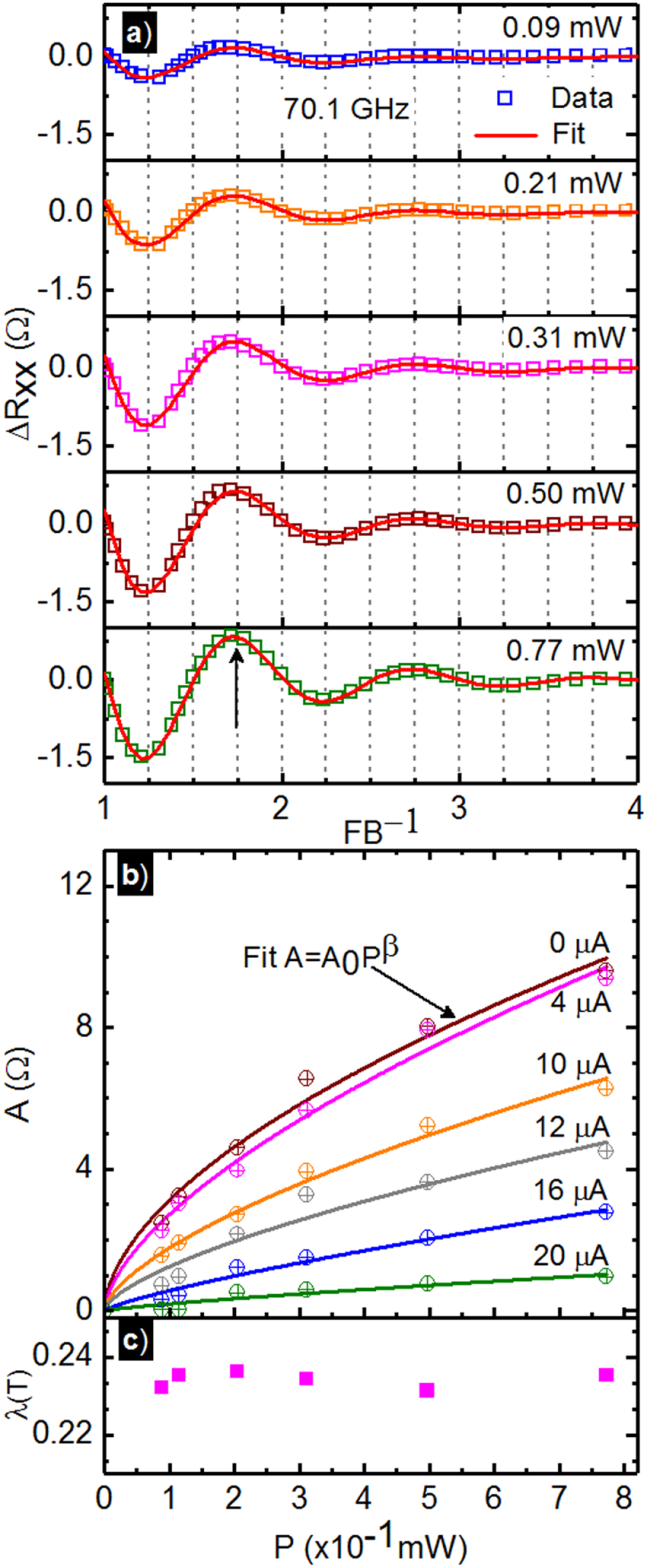
(**a**) This figure exhibits the oscillatory Δ*R*_*xx*_ extracted by subtracting the non-oscillatory magnetoresistance from the experimental data at different microwave power. The plot shows Δ*R*_*xx*_ vs. *FB* ^−1^ at various *P* for *I*_*dc*_ = 0 *μA*. Here, *F* is the magnetoresistance oscillation frequency and *B* is the magnetic field. The symbols represent data while the lines represent fits to exponentially damped sinusoids, see text. (**b**) The magnetoresistance oscillation amplitude, *A*, vs. *P* is shown for different *I*_*dc*_ from 0 *μA* to 20 *μA* (circles). Also shown are the power-law fits, *A* = *A*_0_ *P* ^*β*^ (solid lines). Fit extracted *A*_0_ and *β* are shown in Table 2. (c) The damping constant, *λ*, in the exponentially damped sinusoidal fit of the oscillatory magnetoresistance is plotted vs. *P*.

Figure 3(a) exhibits the experimental oscillatory resistance, Δ*R*_*xx*_ in the range of 0.04 ≤ *B* ≤ 0.16 *T* as symbols vs. the normalized inverse magnetic field scale *FB* ^−1^, where *F* is the magnetoresistance oscillation frequency, for different *P* and *I*_*dc*_ = 0. The Δ*R*_*xx*_ is obtained by subtracting the fit for the non-oscillatory magnetoresistance from the experimental data, as mentioned above. The plot indicates that the oscillatory extrema are shifted by 1/4 unit with respect to integral values on the abscissa scale, confirming a “1/4-cycle” phase shift in the radiation-induced magnetoresistance oscillations^3^. Further, in Fig. 3(a), the height of the oscillatory magnetoresistance peak indicated by the arrow (↑) decreases by ≈80 percent upon reducing the *P* by a factor of eight. The solid lines in red shown in the Fig. 3(a) are the nonlinear least square fits to the data using exponentially damped sinusoids, i.e., ${\rm{\Delta }}{R}^{fit}=-A\,\exp (-\lambda /B)\,\sin \,(2\pi F/B)$ ^3, 5, 12, 15, 25, 28^. Here, *A* is the oscillatory amplitude, *F* is the magnetoresistance oscillation frequency and *λ* is the damping factor. The data fits serve to extract three parameters: *A*, *F* and *λ* ^3, 5, 12, 25, 28^. Since *F* is independent of the radiation-intensity it was fixed to a constant value^25^. The fit indicates *F* = 0.1610 *T* at *f* = 70.1 *GHz*, which suggests that ${m}^{\ast }/m=eF/\mathrm{(2}\pi mf)=0.064$, slightly lower than the standard value, ${m}^{\ast }/m=0.067$ for GaAs/AlGaAs 2DES system. Figure 3(b) shows the oscillatory magnetoresistance amplitude, *A* vs. *P* for different *I*_*dc*_ (circles), along with a fit of the results to *A* = *A*_0_ *P* ^*β*^. Here, the oscillatory resistance amplitude shows a sub-linear growth with increasing *P*, as reported previously. The fit extracted *A*_0_ and *β* are summarized in the Table 2. The table shows that *β* decreases with increasing *I*_*dc*_, a consequence of the fact that the oscillations get smaller with increasing *I*_*dc*_^25, 54^. Finally, Fig. 3(c) shows the fit extracted oscillation damping factor, *λ*, vs. *P*. Here, *λ* = 0.234 ± 0.003 served to fit the entire *P* range.

**Table 2:** Fit parameters obtained for power law fits, see Fig. 3(b), of the amplitude of the oscillatory magneto-resistance induced by photo-excitation at different *I*_*dc*_.

*I*_*dc*_ (*μA*)	*A* (Ω)	*β*.
0	11.5	0.57
4	11.4	0.62
8	7.7	0.64
12	5.6	0.66
16	3.5	0.79
20	1.5	0.81

**Figure 4:**
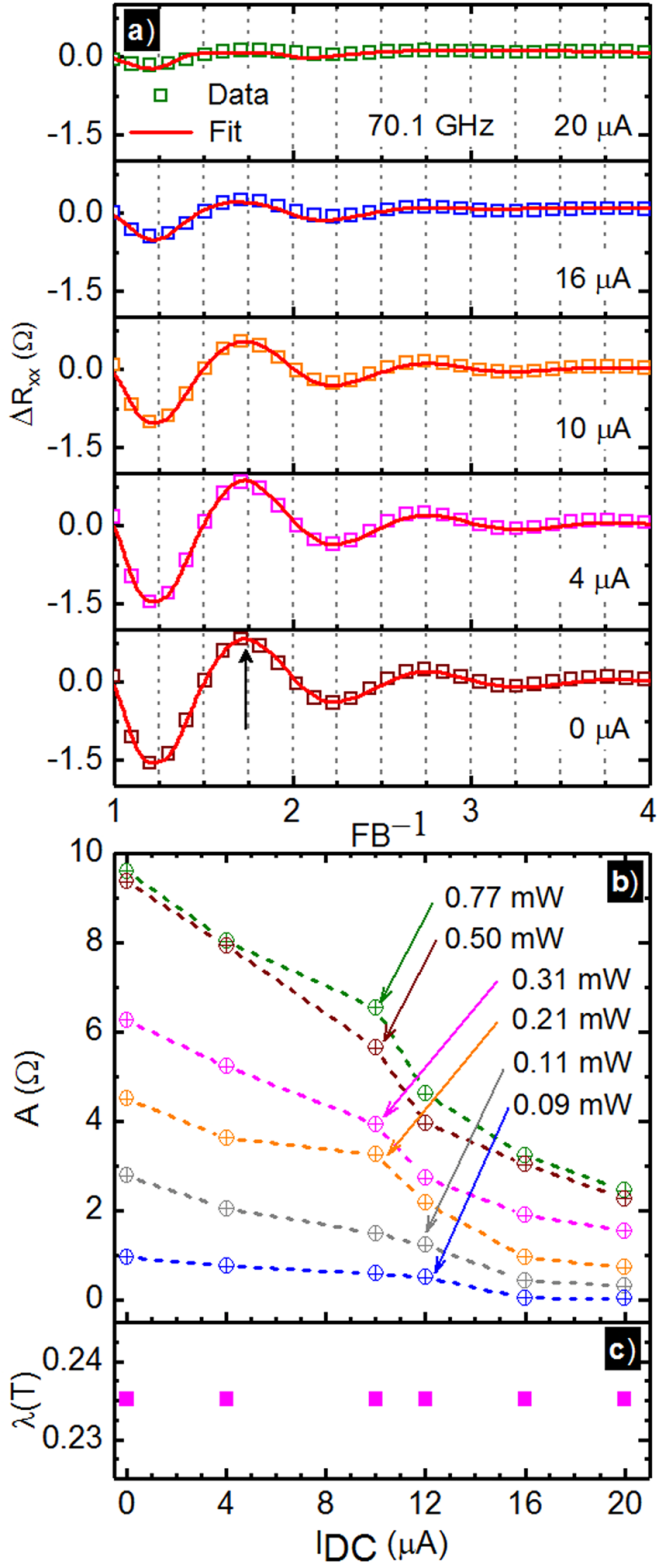
(**a**) This figure exhibits the oscillatory Δ*R*_*xx*_ extracted by subtracting the non-oscillatory magnetoresistance from the experimental data at different *I*_*dc*_. The plot shows Δ*R*_*xx*_ vs. *FB* ^−1^ at various *I*_*dc*_ = 0 *μA* for *P* = 0.77 *mW*. Here, *F* is the magnetoresistance oscillation frequency and *B* is the magnetic field. The symbols represent data while the lines represent fits to exponentially damped sinusoids, see text. (**b**) The fit extracted magnetoresistance oscillation amplitude, *A*, vs. *I*_*dc*_ is shown for different *P* from 0.09 *mW* to 0.77 *mW* (circles). The dashed lines are guides to the eye. (**c**) The damping constant, *λ*, in the exponentially damped sinusoidal fit of the oscillatory magnetoresistance is plotted vs. *I*_*dc*_.

The highlighted oscillatory resistances in Fig. 2(c) have been fit to exponentially damped sinusoids. Figure 4(a) shows Δ*R*_*xx*_ vs. the normalized inverse magnetic field scale *FB* ^−1^, where *F* is the magnetoresistance oscillation frequency, for different *I*_*dc*_ and *P* = 0.77 *mW*, along with data fits to the exponentially damped sinusoids mentioned above. Figure 4(a) shows that the radiation-induced magnetoresistance oscillations are gradually reduced in amplitude with increasing *I*_*dc*_. However, the extrema remain mostly unshifted with increasing *I*_*dc*_. Figure 4(b) show the extracted oscillatory amplitude *A* vs. *I*_*dc*_ at different microwave power levels *P*. From the plot, it is clear that the *A* decreases with *I*_*dc*_ at each *P*. Figure 4(c) shows that a constant damping factor *λ* serves to fit the Δ*R*_*xx*_ for 0 ≤ *I*_*dc*_ ≤ 20 *μA*.

## Discussion

Our recent work has examined a current-tunable giant magnetoresistance in the GaAs/AlGaAs 2D system^80^. This work aimed to study possible interplay between radiation-induced magnetoresistance oscillations and the dc-current induced non-oscillatory giant magnetoresistance, in order to further understand any possible mutual influence between these two effects. Thus, systematic measurements of the *R*_*xx*_ were carried out as a function of both the microwave power *P* (at a constant microwave frequency *f*), and the supplemental dc current *I*_*dc*_.

As a function of *P* at *I*_*dc*_ = 0 *μA*, the canonical “1/4-cycle” shifted radiation-induced oscillations were observed (Fig. 1(a)), the oscillations could be fit with exponentially damped sinusoids (Fig. 3(a)), the oscillatory amplitude increased non-linearly with *P* (Fig. 3(b)), and the damping factor *λ* remained unchanged with *P* (Fig. 3(c))^1, 3, 5–14, 16–19, 21, 22, 24–26, 28–30, 34, 35, 37, 38^.

As a function of *I*_*dc*_ at a fixed *P*, the progresssive increase of *I*_*dc*_ revealed a systematic change in the non-oscillatory giant magnetoresistance (Fig. 1(b)). This nonoscillatory giant magnetoresistance could be successfully fit with a two term Drude model (Fig. 2(a,b))^77^. The fit parameter *σ*_1_ tracked the change in the non-oscillatory magnetoresistance with *I*_*dc*_, see Fig. 2(e). The results show that *σ*_1_ decreases with increasing *I*_*dc*_, sign reversal is observable in *σ*_1_, and the sign reversal correlates with a change from overall positive to overall negative magnetoresistance (cf. Fig. 2(a,e)). Such fits also show that although the non-oscillatory giant magnetoresistance is sensitive to *I*_*dc*_, it is not as sensitive to the microwave power *P*. This latter feature is reflected in the relative invariance of *σ*_1_ vs. *P*, see Fig. 2(d).

As a function of *I*_*dc*_ at fixed *P*, the progressive increase of *I*_*dc*_ also serves to reduce the amplitude of the radiation-induced magnetoresistance oscillations, see Figs 1(b), 2(a,c) and 4(a). For each *I*_*dc*_, the magnetoresistance oscillations at a fixed *P* could be fit with exponentially damped sinusoids, with a constant damping factor *λ*, see Fig. 4(a,c). The magnetoresistance oscillation amplitude dropped monotonically with increasing *I*_*dc*_ at each microwave power *P*, see Fig. 4(b).

This work therefore shows that the *I*_*dc*_ tunable giant magnetoresistance in the GaAs/AlGaAs 2D system follows the multiconduction Drude model even when supplementary radiation-induced magnetoresistance oscillations are induced by microwave photo-excitation of the specimen. Indeed, the radiation-induced magnetoresistance oscillations and the giant magnetoresistance appear separable in the sense that one may fit the non-oscillatory magnetoresistance, proceeding as though the magnetoresistance oscillations do not exist, and remove it from the experimental data, to obtain separated giant magnetoresistance and radiation-induced magnetoresistance oscillations. At the moment, the only observable mutual influence appears to be the reduction in the amplitude of the radiation-induced magnetoresistance oscillations with increased *I*_*dc*_.

A close examination of Fig. 1(b) shows that the reduction in the amplitude of the radiation-induced magnetoresistance oscillations with increasing *I*_*dc*_ proceeds in a curiously asymmetric manner: Increasing *I*_*dc*_ greatly reduces the *R*_*xx*_ at the oscillatory maxima while the effect of the *I*_*dc*_ on the *R*_*xx*_ minima is much smaller. This feature suggests one possible route to understanding the results: In the strong field condition, $\omega \tau \gg 1$, which is satisfied at *B* ≥ 0.001 *T* in such specimens, the *ρ*_*xx*_ is directly proportional to the *σ*_*xx*_, i.e., ${\rho }_{xx}\propto {\sigma }_{xx}/{\sigma }_{xy}^{2}$. This implies that reduced diagonal resistance/resistivity is a consequence of reduced diagonal conductance/conductivity. Thus, one might say that at the oscillatory resistance maxima, the diagonal conductivity is suppressed with increasing *I*_*dc*_ in Fig. 1(b). On the other hand, at the minima, see Fig. 1(b), the relative insensitivity of *R*_*xx*_ to the *I*_*dc*_ indicates that the diagonal conductivity cannot suppressed further by the *I*_*dc*_. One way to understand this feature is to assert that ‘optimal’ microwave photo-excitation reduces the diagonal conductivity to its lowest possible value at the oscillatory minima at a given temperature, and that the *I*_*dc*_ is not very effective in reducing the diagonal conductivity further, below this value. As a consequence, *I*_*dc*_ fails to make a significant change at the oscillatory magnetoresistance minima. On the other hand, at the photo-excited oscillatory magnetoresistance maxima, where photo-excitation serves to enhance the diagonal conductivity above the dark value, the supplemental current can be very effective in reducing the conductivity because there is room to do so and, therefore, the diagonal resistance at the oscillatory maxima is suppressed by the *I*_*dc*_. When, at say *I*_*dc*_ = 20 *μA* in Fig. 1(b), *I*_*dc*_ has its optimal effect, the radiation-induced magnetoresistance oscillations disappear mostly because the supplemental current prevents the oscillatory resistance (conductance) enhancements that occur at the peaks of the radiation-induced magnetoresistance oscillations.

Finally, we note here that prior work by Hatke and co-workers^73^ examined the effect of the dc-drive at cyclotron resonance subharmonics at *f* = 27 GHz. In comparison, we examine the effect of dc-drive at cyclotron resonance harmonics at *f* = 70.1 GHz. Cyclotron resonance subharmonics are not evident at *f* = 70.1 *GHz*. Bykov *et al*.^72^ examined the effect of a dc-drive without microwaves in strong magnetic fields with strong Shubnikov de Haas oscillations, while this work examines the interplay between the dc-drive and the microwave excitation on the magnetotransport.

## Conclusions

In summary, this study shows that a *I*_*dc*_ tunable giant magnetoresistance can coexist with radiation-induced magnetoresistance oscillations in the GaAs/AlGaAs 2D electron system. Further, the two effects are separable and can be separated using a two term Drude multi-conduction model. It appears that the radiation-induced magnetoresistance oscillations have a minimal effect on the current-tunable non-oscillatory magnetoresistance. On the other hand the *I*_*dc*_ responsible for the magnetoresistance produces a progressive and rather asymmetric decrease in the amplitude of the radiation-induced oscillations. The results suggest that the supplemental *I*_*dc*_ serves to produce an overall decrease in the diagonal conductivity, and this serves to reduce and eventually eliminate the conductivity enhancements at the peaks of the radiation-induced oscillatory magnetoresistance. Since dissipative transport in the strong field magnetic limit proceeds by scattering from state to state in the vicinity of the Fermi level, it appears that the *I*_*dc*_ serves to suppress such scattering in the strong field limit.

## Methods

High mobility MBE GaAs/AlGaAs heterostructures were patterned into Hall bars by photolithography. Four terminal electrical measurements were carried out on the Hall bars using low frequency lock-in based techniques with the sample mounted at the end of a cylindrical waveguide, within a variable temperature insert, inside a superconducting solenoid in the *B* ⊥ *I* configuration. Since the 200 *μm* wide Hall bars included voltage probes spaced by 200 *μm*, the effective Length-to-Width (L/W) ratio for the measurements presented here is L/W = 1. The samples were photo-excited via a cylindrical waveguide and the incident power was systematically varied using variable attenuators. The samples were immersed in liquid helium and temperature control was realized by controlling the vapor pressure of liquid helium. The ac- and dc- currents were applied as shown in the inset of Fig. 1(b). The lock-in sourced ac current source was held constant, as a dc current was varied as desired under computer control, at a series of microwave power levels *P*. Typically, magnetic field (*B*) sweeps of the lock-in detected diagonal voltage *V*_*xx*_ were collected at a fixed temperature, *T*, in order to determine ${R}_{xx}={V}_{xx}/{I}_{ac}$.
